# Secular Trend in Age at Menarche and Associated Determinants in the Valencian Population (Spain)

**DOI:** 10.1002/ajhb.70322

**Published:** 2026-09-01

**Authors:** Andrea Beneito, Blanca Sarzo, Raul Beneyto, Reem Abumallouh, Natalia Marin, Oihane Alvarez, Ana Molina‐Barceló, Mercedes Vanaclocha‐Espí, Carmen Freire, Ferran Ballester, Ana Esplugues, Maria‐Jose Lopez‐Espinosa

**Affiliations:** ^1^ Catalan Institute of Health–Camp de Tarragona Primary Care Center Montblanc Montblanc Spain; ^2^ Epidemiology and Environmental Health Joint Research Unit FISABIO–Universitat Jaume I–Universitat de València Valencia Spain; ^3^ Spanish Consortium for Research on Epidemiology and Public Health (CIBERESP) Madrid Spain; ^4^ Department of Nursing, Faculty of Nursing and Chiropody University of Valencia Valencia Spain; ^5^ Cancer and Public Health Area, Foundation for the Promotion of Health and Biomedical Research of the Valencian Region (FISABIO) Valencia Spain; ^6^ Department of Legal Medicine, Toxicology and Physical Anthropology, School of Medicine University of Granada Granada Spain; ^7^ Instituto de Investigación Biosanitaria de Granada (Ibs.GRANADA) Granada Spain

**Keywords:** body mass index, continent of birth, educational level, menarche

## Abstract

**Objective:**

To assess secular trend in age at menarche spanning nearly eight decades and to explore variations in these patterns according to selected sociodemographic and anthropometric determinants in the Valencian Community, Spain.

**Materials and Methods:**

This population‐based study included 417 260 participants born between 1931 and 2008. First, secular trend in age at menarche were assessed using time‐series models across 5‐year birth cohorts for the overall population. Then, 5‐year birth cohorts were combined into two groups based on the data availability for the determinant analyses: an earlier‐born group (1941–1975), whose age at menarche was reported retrospectively in adulthood, and a later‐born group (1991–2005), whose age at menarche was collected during childhood and/or adolescence. Bayesian linear regression models were fitted for each group, adjusting for 5‐year birth cohort and continent of birth in all models, and additionally for educational level in the earlier‐born group and body mass index (BMI) in the later‐born group.

**Results:**

Mean age at menarche decreased by 1.9 years, from 13.1 to 11.1, between the 1931–1935 and 2006–2008 birth cohorts, with a steeper decline after 1975. Compared with European‐born participants, those born in South/Central America (*β* [95% CI]: 0.33 [0.30, 0.36] years) and Africa (0.52 [0.45, 0.58] years) experienced later menarche in the earlier‐born group, whereas those born in South/Central America experienced earlier menarche in the later‐born group (−0.18 [−0.28, −0.09] years). In the later‐born group, lower BMI was associated with later menarche (0.96 [0.74, 1.18] years) whereas higher BMI with earlier onset (−0.53 [−0.57, −0.48] years).

**Conclusion:**

There was a marked decline in age at menarche in the Valencian Community. Factors associated with age at menarche included continent of birth (with cohort‐specific effects) and BMI.

## Graphical abstract

The graphical abstract illustrates changes in age at menarche in the Valencian population (Spain) over nearly eight decades and its association with anthropometric and sociodemographic factors.

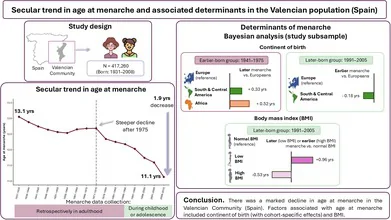

## 1 Introduction

Menarche, defined as the first menstrual period, is a fundamental milestone in female pubertal development that marks the onset of reproductive capacity (Lacroix et al. 2023). It is a late pubertal indicator that occurs at a mean age ± standard deviation (SD) of 12.4 ± 2.5 years, with a typical range of 10–16 years (Lacroix et al. 2023). Its timing is regulated by the endocrine–reproductive system (Lacroix et al. 2023) and is primarily driven by rising estrogen levels (Soriano‐Guillén and Argente 2019; Wood et al. 2019). Beyond biological pubertal processes, menarche involves emotional, psychosocial, cognitive, and physical changes that mark the transition to adolescence (Emmanuel and Bokor 2021; Wood et al. 2019).

At the population level, menarche timing is of public health importance, given the well‐documented associations between earlier menarche and adverse long‐term health consequences, including increased risks of hormone‐related cancers (J. S. Lee et al. 2022; Solbana and Chaka 2023; Soriano‐Guillén and Argente 2019), cardiovascular and metabolic diseases (Charalampopoulos et al. 2014; Lee et al. 2022), mental health disorders (Askelund et al. 2024), and all‐cause mortality (Charalampopoulos et al. 2014; Tamakoshi et al. 2011). In this context, a substantial body of literature has documented a consistent global secular trend toward earlier menarche. Specifically, historical data from Western countries show a decline from ~17 years in the early 19th century to ~13 years by the mid‐20th century (McDowell et al. 2007; Sørensen et al. 2012). In low‐ and middle‐income countries, where ongoing socioeconomic transitions and improvements in nutrition and healthcare are occurring, the age at menarche is also declining (Garenne 2020; Lin et al. 2024; Meher and Sahoo 2024).

These temporal trends suggest that multiple determinants may influence menarche timing, including biological, nutritional, environmental, and socioeconomic factors, with genetic predisposition providing the biological framework (Fernández‐Rhodes et al. 2018; Wang et al. 2024) and the other factors shaping its expression across the life course (Kaplowitz 2008; Sekajová et al. 2022). Historically, improvements in living standards (particularly in healthcare, socioeconomic conditions, and nutrition) have contributed to earlier menarche (Gomula and Koziel 2018), whereas unfavorable conditions have been associated with delays (Gomula and Koziel 2018; Lobstein 2024; Sekajová et al. 2022).

Although the secular decline in menarche age is well documented globally, evidence from Spain remains limited. The available studies containing Spanish data (Cabanes et al. 2009; Mendoza et al. 2010; Onland‐Moret et al. 2005; Sørensen et al. 2012) are scarce and lack recent data, limiting a comprehensive assessment of the magnitude of the secular decline in age at menarche in Spain and preventing conclusions as to whether patterns described in the existing literature have persisted, stabilized, or reversed over time. Moreover, the underlying determinants associated with these patterns of menarcheal timing remain poorly understood in the Spanish context, further highlighting the need for updated, population‐based evidence. To address these gaps, this study examines the secular trend in age at menarche and further explores variations in these patterns according to selected sociodemographic and anthropometric determinants among 417 260 participants from the Valencian Community (Spain) born between 1931 and 2008.

## 2 Methods

### 2.1 Study Population

This study used four data sources from the Valencian Community (Spain): Two population databases and two cohort studies. The first population‐based database comprised women participating in the Valencian Breast Cancer Screening Program (BCSP) (Salas et al. 2012). It included 398 074 participants (born 1931–1974) with data recorded between 1992 and 2019 at ages 45–65 years. The second population‐based database included public healthcare records from the Ambulatory Care Information System of the Valencian Health Agency (SIA‐GAIA) (Generalitat Valenciana. SIA‐GAIA Data Request. Sanitat GVA 2012), comprising 20 381 participants with data from primary care visits (born 1990–2008; data collected 2004–2019 at ages 8–16). Regarding the cohorts, the INMA‐Valencia cohort (Guxens et al. 2012) recruited pregnant women from the Valencia area in 2003–2005, with mothers and offspring followed up prospectively for over 20 years. This database included 132 INMA girls (born 2004–2006; surveys conducted 2013–2021 at ages 9–16 years) and 282 INMA mothers (born 1961–1985; surveyed 2019–2021 at ages 34–57 years). The second cohort, PAPILONG (Abumallouh et al. 2023), comprised socially and economically vulnerable women (i.e., sex workers, NGO‐supported women, and those living in extreme poverty) residing in Valencia province, including 131 women (born 1952–1983; data collected 2021–2023 at ages 39–69 years).

A total of 419 000 participants were gathered across all databases (Figure S1). Of these, 1740 (0.42%) were excluded due to missing data on age at menarche or implausible values (< 8 or > 18 years). The remaining sample included 417 260 participants born between 1931 and 2008 (Tables S1 and S2), who were grouped into 5‐year birth cohorts. The last birth cohort (i.e., 2006–2010) was truncated in 2008 because no data were available beyond that year. The resulting dataset was then used to assess temporal trends in age at menarche.

Analyses of determinants were subsequently restricted to the 45.1% of participants (Figure S1) with complete data for all determinants under study (Table S3). Therefore, participants with missing data on any of the factors studied were excluded of this analysis. Additionally, all individuals from the 1931–1940, 1976–1990, and 2006–2010 (truncated at 2008) birth cohorts were excluded because of limited availability of determinant data and/or small sample sizes. Participants born in North America, Oceania, Asia (for both analyses), and Africa (for the analyses in younger participants only) were also excluded because of small sample sizes (Table S2). After these exclusions, the final sample (*N* = 188 892) was stratified into two groups: the earlier‐born group (participants born 1941–1975; *N* = 177 025) and the later‐born group (participants born 1991–2005; *N* = 11 867). This stratification was driven by differences in the availability of determinant data (as detailed in Section 2.3), which precluded joint analyses. The assessment of age at menarche also differed between the two groups, being reported retrospectively in adulthood for the earlier‐born group but collected during childhood and/or adolescence for the later‐born group.

All study protocols, including two secondary population‐based registries and two datasets collected by the research team, were approved by the DGSP‐CSISP Ethics Committee from Valencia (references numbers: 20180112/03; 20 190 329/3; 20 190 301/09/1; and 20 210 604/10/01). Secondary data were provided anonymized, while investigator‐collected data were anonymized prior to analysis following written informed consent from participants or legal guardians (for minors). All procedures were conducted in accordance with the Declaration of Helsinki.

### 2.2 Age at Menarche

Age at menarche was retrospectively self‐reported in all adult databases, either during routine clinical visits (BCSP) or through interviewer‐administered questionnaires conducted as part of research surveys (INMA mothers and PAPILONG). In the databases of younger participants, age at menarche was recorded in childhood and/or adolescence during routine clinical visits (SIA‐GAIA) or follow‐up visits (INMA girls), based on reports from parents and/or the girls themselves. In INMA girls, when multiple or discrepant reports were available, predefined harmonization criteria were applied by averaging parent and girl reports from the same visit or by retaining the report closest to the event (Freire et al. 2026).

### 2.3 Determinants of Age at Menarche

As data were pooled from multiple sources, the analyses were restricted to variables available in the two large population‐based databases (i.e., year of birth, country of birth, educational level, and body mass index [BMI]). Specifically, year of birth and country of birth (grouped by continent) were available for the entire study population. Educational level was available for participants in BCSP, INMA mothers, and PAPILONG because the younger participants had not yet completed their education. This variable was categorized as primary, secondary, or university education. BMI at or near menarche was available only for participants in SIA‐GAIA and for INMA girls because anthropometric measurements were obtained closer to the time of menarche. BMI z‐scores were calculated from weight and height using the WHO 2006 Child Growth Standards (De Onis et al. 2007) and classified as low (≤ −2 SD), normal (> −2 to ≤ 1 SD), or high (> 1 SD).

### 2.4 Statistical Analysis

First, a time‐series model was used to characterize the overall pattern of age at menarche and its secular trend in the full sample of participants (*N* = 417 260). Although year of birth was the most precise variable available, some years were only represented by a small number of women, which limited the stability of yearly estimates. Therefore, participants were grouped into 5‐year birth cohorts to reduce variability. Stationarity of the 5‐year series was assessed using the augmented Dickey‐Fuller test (Dickey and Fuller 1979).

Second, analyses of determinants were conducted in a reduced subsample (*N* = 188 892), after the exclusions previously described in Section 2.1. To assess potential selection bias, logistic regression models were used to compare individuals included in the determinant analyses with those excluded, and *p* value were calculated through the likelihood ratio test (LRT). As described above, the study population was stratified into participants born between 1941 and 1975 (*N* = 177 025) and participants born between 1991 and 2005 (*N* = 11 867) because different determinants were available for each group. Specifically, age at menarche for participants in the earlier‐born group was modeled as a function of birth year, birth continent, and educational level, whereas for the later‐born group, models included birth year, birth continent, and BMI (*z*‐scores). For these analyses, Bayesian linear models were fitted using the JAGS software (Plummer 2003). Age at menarche was assumed to have a Normal distribution with mean *μ* and standard deviation *σ*. The mean (*μ*) was modeled as a function of the covariates, whereas *σ* was assigned a Uniform distribution ranging from 0 to 10. The final approximated posterior distributions were obtained using three chains and 3000 iterations, discarding the first 300 as burn‐in. Convergence was assessed via the Brooks–Gelman–Rubin (BGR) statistic (Gelman et al. 2013). Results were presented as posterior means (*β*) and 95% credible intervals (95% CI) from the marginal posterior distribution of model parameters associated with each covariate. For those parameters, non‐informative prior distributions were also used; particularly, Normal prior distributions with mean 0 and precision 0.001.

Finally, several sensitivity analyses were performed to examine the robustness of the findings. First, to assess the potential impact of restricting the analysis to a subsample with complete information on determinants, the secular trend analyses were repeated in this subset and the resulting curves were compared with those from the full study population (time‐series model) by overlaying them in the same graph. Second, to address the potential overlap between data sources in the secular trend analyses, participants from the research cohorts who were born within the years covered by the population‐based databases were excluded. Specifically, 217 INMA mothers (born: 1961 to 1974) and 110 PAPILONG participants (born: 1952 to 1974) were excluded due to possible overlap with the BCSP dataset, whereas 112 INMA girls (born: 2004 to 2006) were excluded due to potential overlap with the SIA‐GAIA dataset. Third, secular trend analyses were conducted separately for each data source to assess whether the observed trends were consistent across the databases (BCSP, PAPILONG, INMA mothers, INMA girls, and SIA‐GAIA).

Statistical analyses were performed with R software version 4.4.2 (R Core Team 2026).

## 3 Results

### 3.1 Study Population

The study comprised 417 260 participants (Table S1), 91.0% of whom were born in Europe, whereas South/Central America (6.89%), Africa (1.45%), Asia (0.54%), and North America/Oceania (0.10%) accounted for much smaller proportions of the study population (Tables 1 and S2). Of the total population, 95.1% were participants born between 1931 and 1985, while the remainder were born between 1990 and 2008. Among participants born between 1931 and 1985, 22.3% had completed secondary education and 15.4% had a university education. Among participants born between 1990 and 2008, 0.97% and 43.5% had low and high BMI, respectively (Table 1).

**TABLE 1:** Characteristics of the study population (1931–2008).

Variable	Overall population born 1931–2008 (*N* = 417 260)	Participants born 1931–1985 (*N* = 396 747)	Participants born 1990–2008^a^ (*N* = 20 513)
Age at menarche (years)	12.6 ± 1.57	12.6 ± 1.57	11.7 ± 1.19
Continent of birth			
Europe	194 959 (91.0)	182 884 (90.9)	12 075 (93.4)
South/Central America	14 755 (6.89)	14 070 (6.99)	685 (5.30)
Africa	3101 (1.45)	3009 (1.50)	92 (0.71)
Asia	1165 (0.54)	1094 (0.54)	71 (0.55)
North America and Oceania	216 (0.10)	211 (0.11)	5 (0.04)
BMI (z‐scores)			
Low BMI	—	—	192 (0.97)
Normal BMI	—	—	11 028 (55.6)
High BMI	—	—	8623 (43.5)
Educational level			
Primary education	—	209 736 (62.3)	—
Secondary education	—	74 918 (22.3)	—
University education	—	51 961 (15.4)	—

*Note:* “–” indicates the absence of observations in the corresponding category. Sample size is presented as *N*, categorical variables as *N* (%), and continuous variables as mean ± SD or range. This table shows the available information for the full population used in the time‐series analysis. For the corresponding data for participants included in the Bayesian analysis of determinants, see Tables S4 and S5. Sample size varies across variables due to differences in data availability. Educational level and BMI were available only for the earlier‐born and later‐born groups, respectively. North America and Oceania were combined due to low numbers.

^a^ Although birth cohorts were defined as 5‐year intervals in this study, only the birth years for which data were available (1990–2008) are shown for the later‐born group in this table, as the 1986–1990 cohort included only births from 1990 and the 2006–2010 cohort only births up to 2008. BMI: body mass index, based on WHO age‐ and sex‐specific z‐scores (De Onis et al. 2007), classified as low (≤ −2 standard deviations [SD]), normal (> −2 to ≤ 1 SD), or high (> 1 SD).

Given differences in determinant data availability, a subsample comprising 45.1% of the study population was included in the determinant analyses. As shown in Table S3, the availability of key variables varied markedly across cohorts. Among participants born 1931–1985, continent‐of‐birth data were initially unavailable (0% completeness for the 1931–1935 birth cohort) and increased progressively across birth cohorts, reaching near‐complete or full coverage among those born between 1966 and 1985. Among participants born 1990–2008, completeness was more variable (25.5%–77.5%), with lower coverage in the younger cohorts. Educational level data were only available for the older participants (completeness range: 73.5%–100%), whereas BMI data were only available for the younger participants (completeness range: 93.8%–98.6%).

Compared with excluded participants (*N* = 228 368), those included in the determinant analyses (*N* = 188 892) showed no differences in age at menarche (*p* = 0.111). However, included participants were more likely to have been born in Europe, had higher educational attainment, and showed small but statistically significant differences in BMI distribution compared with excluded participants (all *p* < 0.05) (Table S4).

### 3.2 Secular Trend of Age at Menarche

**FIGURE 1:**
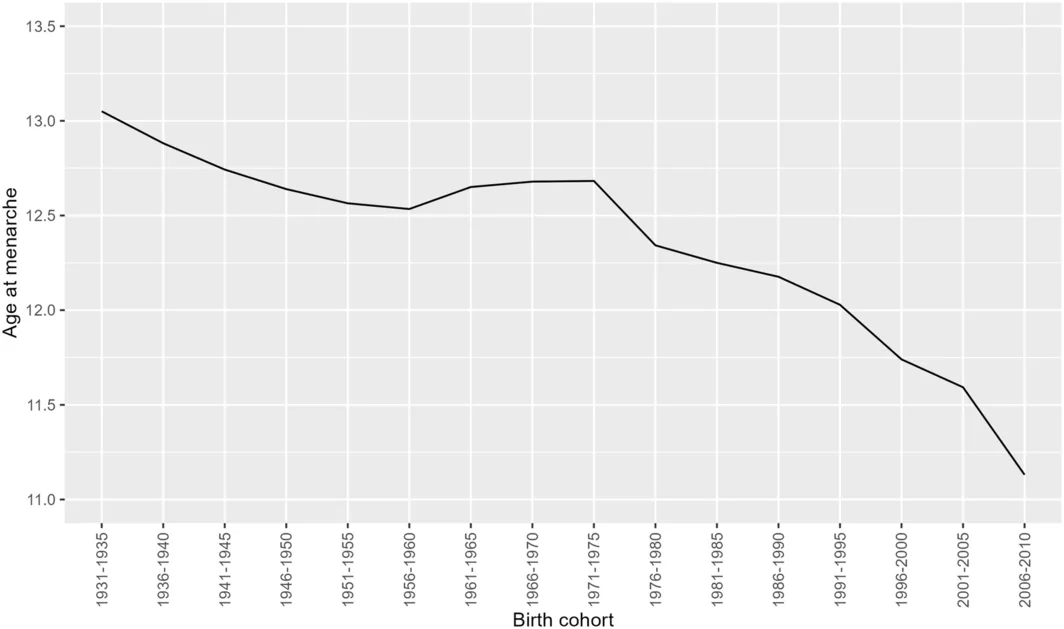
Secular trend in age at menarche by 5‐year birth cohort from 1931 to 2008 (*N* = 417 260). For the most recent 5‐year birth cohort (2006–2010), data availability was limited to births up to 2008.

The mean age at menarche ± SD was 12.6 ± 1.57 years for the overall population (1931–2008): 12.6 ± 1.57 years for participants born 1931–1985 and 11.7 ± 1.19 years for those born 1990–2008 (Table 1). Analysis by 5‐year birth cohort showed a gradual decrease in age at menarche over time. The highest mean ± SD was 13.1 ± 1.65 years in the earliest birth cohort (1931–1935), whereas the lowest was 11.1 ± 0.84 years in the most recent cohort (2006–2010), which was truncated at 2008 because data were unavailable beyond that year. However, the decline in mean age at menarche was not linear across birth cohorts, with an initial gradual decline until 1960, followed by a period of relative stability (1961–1975) and a more pronounced decrease from 1976 onward (Figure 1 and Table S5).

### 3.3 Age at Menarche and Determinants

**FIGURE 2:**
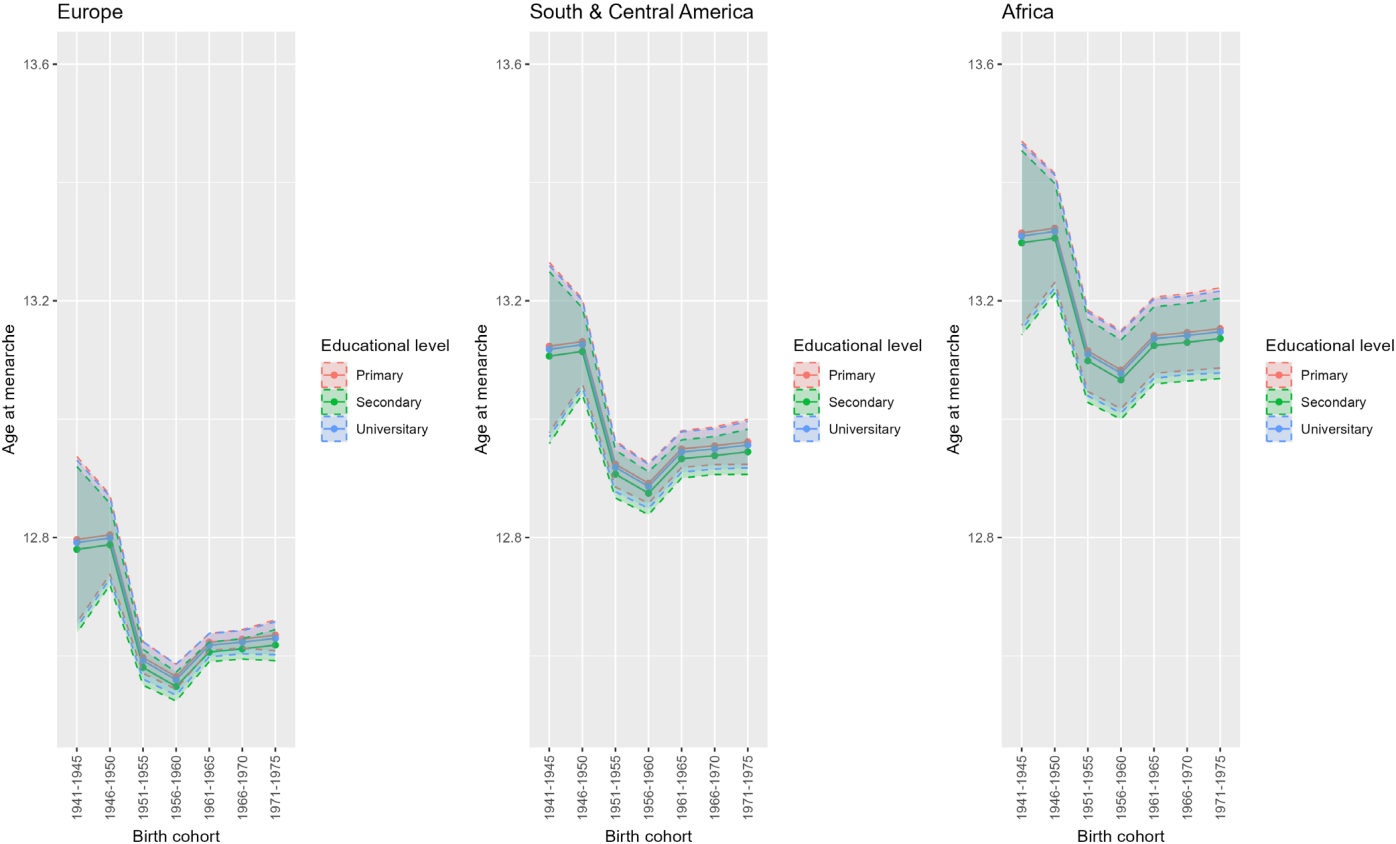
Posterior mean and 95% credible interval (95% CI) of age at menarche for the earlier‐born group (1941–1975; *N* = 177 025), by 5‐year birth cohort, continent of birth, and educational level.

Among the women included in the Bayesian analysis (*N* = 177 025), age at menarche decreased slightly across birth cohorts. The posterior mean age (*β* [95% CI]) declined from 12.8 (12.7–12.9) to 12.6 (12.6–12.7) years for participants born in 1941–1945 and 1971–1975 (Table 2). Among the earlier‐born group, participants born in South/Central America and Africa had a later age at menarche than women born in Europe (0.33 [0.30, 0.36] and 0.52 [0.45, 0.58] years, respectively). Compared with women with primary education, those with secondary and university education reached menarche modestly earlier, with estimates of −0.02 (−0.03, −0.01) years and −0.01 (−0.03, 0.01) years, respectively, although the association was not relevant among university‐educated women (Table 2 and Figure 2). Regarding convergence, all parameters had a BGR statistic of 1 and the effective sample size ranged from 2400 to 8100.

**TABLE 2:** Posterior mean (*β*) and 95% credible interval (95% CI) for model parameters in the earlier‐born group (1941–1975; *N* = 177 025).

Variable	*β* (95% CI)
Year of birth (range)	
1941–1945	12.8 (12.7, 12.9)
1946–1950	12.8 (12.7, 12.9)
1951–1955	12.6 (12.6, 12.6)
1956–1960	12.6 (12.5, 12.6)
1961–1965	12.6 (12.6, 12.6)
1966–1970	12.6 (12.6, 12.6)
1971–1975	12.6 (12.6, 12.7)
Continent of birth	
Europe	Ref
South/Central America	0.33 (0.30, 0.36)
Africa	0.52 (0.45, 0.58)
Educational level	
Primary education	Ref
Secondary education	−0.02 (−0.03, −0.01)
University education	−0.01 (−0.03, 0.01)

*Note:* Ref indicates the reference category. Participants born in the 5‐year birth cohorts 1931–1940 and 1976–1985 were excluded from the determinant analyses due to insufficient sample size. Analyses were conducted among women with complete data on the three determinants.

**FIGURE 3:**
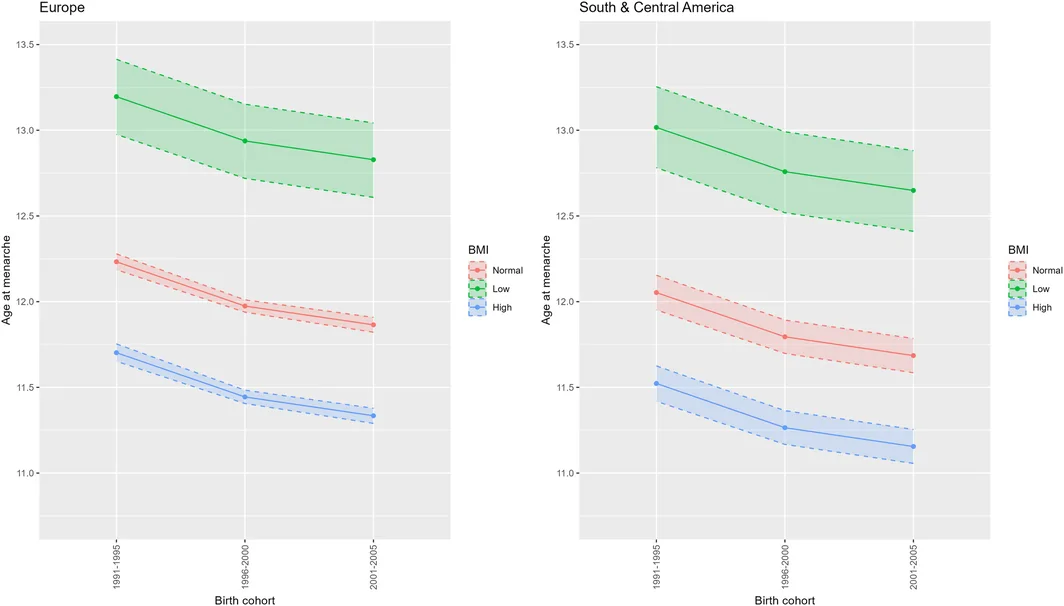
Posterior mean and 95% credible interval (95% CI) of age at menarche for the later‐born group (1991–2005; *N* = 11 867), by 5‐year birth cohort, continent of birth, and BMI. BMI, body mass index, based on WHO age‐ and sex‐specific *z*‐scores (De Onis et al. 2007), classified as low (≤ −2 standard deviations [SD]), normal (> −2 to ≤ 1 SD), or high (> 1 SD).

Among the later‐born group (*N* = 11 867), those born in 1991–1995 had a slightly higher age at menarche than those born in 1996–2000 and 2001–2005 (12.2 [12.2, 12.3]; 12.0 [11.9, 12.0]; and 11.9 [11.8, 11.9] years, respectively). Participants from South/Central America had a lower age at menarche than those born in Europe (−0.18 [−0.28, −0.09] years). Compared with normal BMI, low BMI was associated with later menarche (0.96 [0.74, 1.18] years), and high BMI with earlier menarche (−0.53 [−0.57, −0.48] years) (Table 3 and Figure 3). Regarding convergence, all parameters had a BGR statistic of 1 and the effective sample size ranged from 6800 to 8100.

**TABLE 3:** Posterior mean (*β*) and 95% credible interval (95% CI) for model parameters in the later‐born group (1991–2005; *N* = 11 867).

Variable	*β* (95% CI)
Year of birth (year range)	
1991–1995	12.2 (12.2, 12.3)
1996–2000	12.0 (11.9, 12.0)
2001–2005	11.9 (11.8, 11.9)
Continent of birth	
Europe	Ref
South/Central America	−0.18 (−0.28, −0.09)
BMI (z‐scores)	
Normal BMI	Ref
Low BMI	0.96 (0.74, 1.18)
High BMI	−0.53 (−0.57, −0.48)

*Note:* BMI: body mass index, based on WHO age‐ and sex‐specific z‐scores (De Onis et al. 2007), classified as low (≤ −2 standard deviations [SD]), normal (> −2 to ≤ 1 SD), or high (> 1 SD). Ref indicates the reference category. Participants in the 1986–1990 and 2006–2010 birth cohorts were excluded because limited data coverage resulted in insufficient sample sizes. Specifically, all individuals in the 1986–1990 cohort were born in 1990, and no data were available for births in 2009–2010 within the 2006–2010 cohort.

### 3.4 Sensitivity Analyses

Sensitivity analyses supported the robustness of the observed secular trends. First, secular trends were similar in the full sample (*N* = 417 260) and the subsample with complete determinant data (*N* = 188 892) (Figure S2). Furthermore, the results were materially unchanged after excluding potentially overlapping participants (*N* = 327 and 112 in the older and younger birth groups, respectively; data not shown). Finally, analyses stratified by data source showed a clear secular decline in age at menarche. Although the overall pattern was broadly consistent across data sources in women, some discrepancies were observed, particularly in the PAPILONG cohort, which showed more pronounced fluctuations. Notably, the most pronounced fluctuations in PAPILONG coincided with 5‐year birth cohorts with very small sample sizes, suggesting that these fluctuations may partially reflect random variation. In contrast, the estimates for the younger group were consistent across the two data sources (i.e., INMA girls and SIA‐GAIA) (Figure S3).

## 4 Discussion

In our study of 417 260 participants (born 1931–2008) residing in the Valencian Community, age at menarche declined by approximately 1.9 years over nearly eight decades, from 13.1 to 11.1 years. The association with continent of birth varied by birth group: later menarche among South/Central American and African participants in the earlier‐born group, and earlier onset among South/Central American participants in the later‐born group. Additionally, higher BMI was associated with earlier age at menarche in the later‐born group. Associations with educational level were modest in magnitude and did not show a clear, statistically significant gradient across categories in women.

### 4.1 Secular Trend in Age of Menarche

Although age at menarche declined over the study period, analysis by 5‐year birth cohort showed that the secular decline was gradual during the early decades. Specifically, the mean age at menarche fell from 13.1 years (born 1931–1935) to 12.5 years (born 1956–1960). This pattern may reflect historical improvements in nutrition, living conditions, and healthcare in Spain between 1930 and 1960, although these trends were likely disrupted by the Spanish Civil War (1936–1939) and its aftermath (Cabanes et al. 2009; Marrodán et al. 2012). These findings are consistent with two studies conducted in other Spanish regions (Cabanes et al. 2009; Mendoza et al. 2010), which also reported a gradual decline in age at menarche from the mid‐1920s to 1960. Additionally, a similar generalized decline throughout the first half of the 20th century was observed for Spain in two studies including nine European (Onland‐Moret et al. 2005) and 11 Western (Sørensen et al. 2012) countries. Interestingly, across these four studies, mean age at menarche (visually estimated from published figures in some cases) declined from approximately 14.8 years in the 1930s to 12.2 in the 1960s in Spain (Cabanes et al. 2009; Mendoza et al. 2010; Onland‐Moret et al. 2005; Sørensen et al. 2012).

After the initial decline, age at menarche stabilized in women born between 1961 and 1975 (mean age: 12.7 years). This plateau may partly reflect a period in Spain without major improvements in nutrition and childhood health, before increases in adiposity and lifestyle changes associated with the nutritional transition became more pronounced (Cabanes et al. 2009; Marrodán et al. 2012). Two Spanish studies observed a broadly stable trend with only a slight increase over time (born 1959–1964: mean age 12.5–12.6 years (Onland‐Moret et al. 2005); and born 1959–1962: mean age 12.8–13.0 years (Cabanes et al. 2009)). Regarding other European countries, a Norwegian study reported a clear plateau of a decade (1955–1964: 13.2–13.2 years) (Gottschalk et al. 2020), whereas longer plateaus were observed in Portugal (1950–1979: 12.5–12.5 years) (Queiroga et al. 2020), the United Kingdom (1945–1989: 12.6–12.6 years) (Morris et al. 2011), and Japan (1960–1980: 12.3–12.2 years) (Hosokawa et al. 2012), coming some of the aforementioned data from visual inspection of figures in the respective articles.

From 1976 to 2008, age at menarche showed a marked overall decline in our study (12.3 to 11.1 years), despite a brief increase in the mid‐1980s that should be interpreted cautiously due to the small number of available observations between 1976 and 1990 (*N* = 137). The decline that resumed in cohorts born after the mid‐1970s coincided with the Spanish democratic transition, a period characterized by rapid socioeconomic changes and an intensified nutritional transition (Marrodán et al. 2012). This overall decrease, though much more pronounced in our study, aligns with declines reported in Portugal (born 1970–2000: 12.5–12.0 years) (Queiroga et al. 2020), Taiwan (1975–1989: 12.9–12.2 years) (Lin et al. 2024), and the United Kingdom (1985–1993: 12.6–12.3 years) (Morris et al. 2011). A multicounty study including Spanish data also reported small but statistically significant decreases of 2.5–4 months in age at menarche in recent decades and suggested that the decline has slowed since the 1960s in high‐income settings (Sørensen et al. 2012), whereas no evidence of such a slowdown was observed in our study.

Overall, we observed an average reduction of approximately 1.9 years in age at menarche over nearly eight decades, equivalent to about 0.1 years (36 days) per 5‐year birth cohort, or approximately 72 days per decade. When analyzed separately by birth group, the approximate reduction in age at menarche was 0.08 years (29 days) per 5‐year birth cohort among participants born between 1931 and 1985, and 0.26 years (95 days) per 5‐year birth cohort among participants born between 1990 and 2008. This decline in earlier cohorts (i.e., born between 1931 and 1985) is broadly comparable, albeit slightly lower in our study, with those reported in two previous Spanish studies conducted in other regions that reported decreases of approximately 36.5 days per 5‐year birth cohort between 1940 and 1980 (Mendoza et al. 2010) and 48 days per 5‐year birth cohort between 1925 and 1962 (Cabanes et al. 2009), with no comparable Spanish data from the 1980s onwards. Among studies from other Western countries, a study across nine European countries (Onland‐Moret et al. 2005) reported a decline of ~44 days per 5‐year birth cohort between 1918 and 1964 (country‐specific estimates ranged from 18 days in the UK to 58 days in Spain and Germany), suggesting that the decline observed in our study for earlier cohorts was more modest than that reported for Spain in that multi‐country study. Among European single‐country studies, a Portuguese study reported estimates nearly identical to ours for the overall study period (decline of ~31.1 days per 5‐year birth cohort between 1920 and 2000) (Queiroga et al. 2020), whereas a Norwegian study found a less pronounced decrease than that observed in our earlier‐born group (~13 days per 5‐year birth cohort among participants born 1936–1964) (Gottschalk et al. 2020). Outside Western countries, a large African study reported findings similar to those observed in our older birth group, reporting an average decline of 29 days per 5‐year birth cohort among participants born 1935–1965, with steeper declines in sub‐Saharan Africa and more modest or heterogeneous trends elsewhere (Garenne 2020). In Latin America, the decline was markedly steeper in Colombia (born 1941–1989: ~100 days per 5‐year birth cohort) (Villamor et al. 2009) than in Mexico (born < 1940 to 1980: 27 days per 5‐year birth cohort), the latter being more comparable with our results (Marván et al. 2020). In Asia, studies conducted in China (Ma et al. 2023; Meng et al. 2017), Taiwan (Lin et al. 2024), Korea (D. H. Lee et al. 2024), India (Meher and Sahoo 2024), and Indonesia (Wahab et al. 2018) among women born in the mid‐ to late 20th century reported a secular decline of ~0.035 to 0.36 years per 5‐year birth cohort (~13–130 days) among women born 1927–2010.

Differences in the magnitude and pace of the decline in age at menarche across countries may partly reflect variation in socioeconomic development, nutritional status, and environmental conditions, which are known to influence pubertal timing across diverse populations worldwide (Euling et al. 2008; Leone and Brown 2020; Yermachenko and Dvornyk 2014). Moreover, the temporal scope of the studies may contribute to these discrepancies, as secular changes in living conditions over the last century are unlikely to have occurred at the same rate across populations. In addition, differences in data collection methods, particularly the use of retrospectively reported menarche in adult women versus prospectively or contemporaneously reported data in adolescents, may have introduced recall bias and contributed to the observed variability. This potential source of bias is also relevant to our study, which includes both adult women who reported menarche retrospectively and girls assessed closer to the time of menarche. Nevertheless, menarche is a salient life event that tends to be remembered with considerable accuracy even decades later (Lundblad and Jacobsen 2017). Indeed, several studies have reported moderate to strong agreement between adult recall and prospectively recorded adolescent data, relatively small recall errors, and high consistency in repeated assessments over time (Cooper et al. 2006; Koprowski et al. 2001; Lundblad and Jacobsen 2017; Must 2002; Tehrani et al. 2010), supporting the reliability of self‐reported age at menarche across different reporting contexts. However, recall accuracy may vary according to educational level (Cooper et al. 2006), reproductive history (Cooper et al. 2006), and age at menarche itself, with late menarche tending to be recalled as occurring somewhat earlier (Lundblad and Jacobsen 2017; Must 2002).

### 4.2 Determinants Associated With the Secular Trend in Menarche

In the present study, birth continent was associated with age at menarche, although the observed patterns differed between both birth groups, who represent distinct birth cohorts. Specifically, participants belonging to the older birth group (i.e., born ≤ 1975) and born in South/Central America or Africa tended to report a slightly later age at menarche compared to European women, whereas among the younger birth group (i.e., born from the 1990s onward), those from South/Central America showed an earlier onset of menarche, while no data were available for those from Africa. This reversed pattern among generations may reflect the complex interplay between biological factors and the socioeconomic and environmental context influencing pubertal development among migrant women (Sekajová et al. 2022; Wang et al. 2024). In the Spanish context, following the country's transition to a destination for immigration since the 1990s (Finotelli and Rinken 2023), one possible interpretation is that older foreign‐born cohorts likely experienced menarche in their countries of birth, whereas younger cohorts may have migrated earlier in life and thus experienced menarche under Spanish nutritional and health conditions. However, in the absence of data on age at migration, duration of residence in Spain, and early‐life socioeconomic or environmental conditions, these interpretations should be treated with caution. An additional consideration is that the two groups represent distinct birth cohorts that were analyzed using different sets of variables and different approaches to assessing age at menarche (retrospective in adult women versus collected closer to adolescence in the younger cohort), which may partly explain the contrasting associations with continent of birth.

Analyses of educational level were restricted to the older birth group because younger participants had not yet completed their education at the time of data collection. Associations were modest and did not show a clear gradient across categories. Only secondary education showed evidence of an association with earlier menarche compared with primary education. The association between age at menarche and education may reflect shared socioeconomic conditions rather than causality, as education is often used as a proxy for socioeconomic status, yielding inconsistent results across studies. For example, studies in the United States (Wang et al. 2024) and the United Kingdom (Morris et al. 2011) reported earlier menarche in lower socioeconomic groups, whereas Mexican data (Marván et al. 2020) showed the opposite pattern. In addition, although not measured in our study, childhood social and family environments warrant consideration because of their potential role in pubertal timing and development. For example, adverse psychosocial conditions during childhood, including father absence, family disruption, and other adverse childhood experiences have been associated with earlier menarche (Deardorff et al. 2014; Glass et al. 2022; Zhang et al. 2019). However, this association may be attenuated by extended family and community support networks (Deardorff et al. 2014).

In our study, higher BMI in participants from the later‐born group were associated with earlier menarche, whereas those with lower BMI tended to reach menarche later. However, these analyses could not be conducted in participants from the earlier‐born group because BMI measurements at or near menarche were unavailable. Our findings are consistent with previous studies (Lee et al. 2024; Meng et al. 2017) and align with prior literature suggesting that adiposity is an important determinant of pubertal timing (Kaplowitz 2008). Several biological mechanisms may underlie this association: increased body fat is linked to higher leptin concentrations, altered insulin sensitivity, and enhanced peripheral estrogen production, which may promote earlier activation of the hypothalamic–pituitary–gonadal axis and accelerate pubertal progression, whereas low adiposity may delay these processes because of insufficient energy availability and hormonal signaling (Kaplowitz 2008; Kythreotis et al. 2025). In this context, the increasing prevalence of overweight and obesity among children in Spain in recent decades (Bravo‐Saquicela et al. 2022) may represent one of several factors contributing to the earlier menarche observed in our population.

BMI is also closely associated with lifestyle factors, including nutritional quality and physical activity during childhood and adolescence (Lozano et al. 2025), which may further influence pubertal timing. Although not directly assessed in our study, these factors are closely intertwined with adiposity and may help explain the observed associations. In particular, nutritional quality may play a role in the timing of menarche through its effects on energy balance, growth, and metabolic regulation (Fitch et al. 2026). A recent study reported that girls with healthier dietary patterns experienced later menarche, whereas those consuming more pro‐inflammatory diets reached menarche earlier, and these associations remained after adjustment for BMI and height (Davis et al. 2025), suggesting that dietary quality may influence menarcheal timing independently of adiposity. Likewise, physical activity may influence menarche through its effects on body composition, energy balance, and endocrine regulation (Fitch et al. 2026; Kehm et al. 2024). Higher levels of physical activity during childhood have generally been associated with later age at menarche, although the magnitude of this association appears to depend on the timing, intensity, and duration of exercise (Fitch et al. 2026; Kehm et al. 2024).

Another potential determinant, also related to adiposity, is exposure to environmental obesogens such as endocrine‐disrupting chemicals (EDCs) (Montazeri et al. 2023), which may influence pubertal timing through multiple biological pathways, including effects on adiposity and hormonal regulation (J. E. Lee et al. 2019; Supornsilchai et al. 2016). Although exposure to EDCs could not be examined in the present study because these data were unavailable in the large population‐based databases, previous research conducted within one of the cohorts included in this study (the INMA cohort) has identified associations between several EDCs (including organochlorine compounds, phthalates, phenols, parabens, non‐persistent pesticides, perfluoroalkyl substances, and metals) and pubertal timing (Beneito, Carrizosa, et al. 2026; Castiello et al. 2023; Freire et al. 2024; Freire et al. 2026; Lopez de Calle et al. 2026; Sarzo et al. 2022), although the direction of these associations varied across compounds, with both earlier and later pubertal timing reported.

### 4.3 Limitations, Strengths, and Health Implications

This study has several limitations. First, information on determinants was limited to year of birth, continent of birth, BMI, and educational level, as only these variables were available in the two large databases, thereby precluding the inclusion of additional variables available in the smaller cohorts. Consequently, several potentially important factors, such as migration history (e.g., age at migration or duration of residence in Spain), childhood socioeconomic conditions, nutritional quality, physical activity, psychosocial stress, or environmental exposures (including EDCs) could not be examined. Second, differences in determinant availability across data sources (year of birth and continent of birth for both groups, educational level for the earlier‐born group, and BMI for the later‐born group) required separate analyses in both birth groups. Age at menarche was also assessed differently in the two groups: retrospectively in adult women, with potential for recall error, and contemporaneously in children and adolescents. As a result, direct comparisons between groups should be interpreted with caution. Third, only individuals with complete information on all predictors were included in the determinant analyses. Compared with excluded participants, those included differed in continent of birth, educational level, and BMI, which may have limited the representativeness of the analytic sample. However, these differences were not accompanied by differences in age at menarche and could be partly explained by data availability, small sample sizes, and historical factors. In particular, participants from North America, Oceania, and Asia in both groups, as well as from Africa in the younger birth group, were excluded because of insufficient sample sizes. Furthermore, country‐of‐birth information was not systematically collected in the earliest cohorts, resulting in the exclusion of many women born during these periods. Given the historical context of Spain, where access to secondary and university education was more limited among older generations (Blanco and Luis Hernández Huerta 2012), the exclusion of these earlier cohorts likely contributed to the higher educational attainment observed among included participants. Also, although BMI differed between included and excluded participants, the overall distributions were similar. Fourth, the most recent birth cohort (2006–2010) was only partially represented, as birth data were available only up to 2008. In addition, no data was available for participants born after 2008, limiting our ability to assess more recent trends. Finally, the SIA‐GAIA database recorded age at menarche as free text, which may have affected data completeness and consistency, potentially leading to an overrepresentation of cases of precocious or delayed puberty.

This study also has several strengths. First, the large, population‐based sample (*N* = 417 260), spanning 1931–2008, provides substantial statistical power and a unique opportunity to assess long‐term generational changes in pubertal timing within a defined socioeconomic and cultural context. Second, the use of multiple complementary data sources, including population‐based registries and cohort studies, enhances the representativeness and validity of the findings. Third, the inclusion of women from diverse origins provides valuable insights into regional and ethnic differences in age at menarche within the Valencian Community. Finally, the study explores variations in age at menarche according to key anthropometric and sociodemographic determinants, including factors that may be amenable to intervention, such as BMI. Overall, this study advances our understanding of both secular trends and the determinants of age at menarche, helping to inform future prevention and public health strategies.

With regard to the health implications, the decline of almost two‐years in age at menarche observed over nearly eight decades in our study may be both biologically and clinically meaningful, with potential implications for women's health across the life course. Beyond a reduction in the mean age at menarche, a shift of this magnitude may also lead to a greater proportion of girls experiencing early or very early menarche (Wang et al. 2024). Prior literature has linked earlier menarche with an increased risk of mental health problems (Askelund et al. 2024). In adulthood it has been associated with a range of cardiometabolic disorders, including obesity, insulin resistance, Type 2 diabetes, hypertension, and metabolic syndrome, as well as other conditions such as endometriosis, gestational diabetes, asthma, and hormone‐related cancers (breast, endometrial, and ovarian) (J. S. Lee et al. 2022). Additionally, a previous meta‐analysis showed that women with menarche before 12 years of age have a higher risk of all‐cause mortality than those with later menarche (Charalampopoulos et al. 2014). Taken together, these observations suggest that the secular decline in age at menarche may have important implications for women's health and warrants continued monitoring from a public health perspective.

## 5 Conclusion

Our findings indicate a 1.9‐year decrease in age at menarche among participants from the Valencian Community who were born between 1931 and 2008, consistent with global evidence of a long‐term downward trend. This decline was gradual among the earliest cohorts, followed by a temporary stabilization between 1960 and 1975, after which the decline resumed. The mean decrease was approximately 0.1 years (36 days) per 5‐year birth cohort, equivalent to about 72 days per decade, and the mean age at menarche dropped below 12 years in the youngest generations. Our findings also suggest that pubertal timing is a multifactorial process, with associations observed for continent of birth and BMI. Associations with continent of birth varied across birth cohorts. Compared with European‐born participants, those born in South/Central America or Africa had a later age at menarche in the earlier‐born group, whereas the opposite pattern was observed among those born in South/Central America in the later‐born group. These findings should be interpreted with caution because of potential cohort effects, differences in data availability and methods of assessing age at menarche, as well as the lack of information on migration history and early‐life conditions. Higher BMI was associated with earlier menarche, suggesting that childhood obesity may play an important role in pubertal timing and underscoring its potential importance for public health strategies.

## Author Contributions

**Andrea Beneito:** conceptualization; data curation; formal analysis; investigation; methodology; writing – original draft. **Blanca Sarzo:** data curation; formal analysis; supervision; methodology; writing – original draft; writing – review and editing. **Raul Beneyto:** data curation; formal analysis; methodology; writing – original draft; writing – review and editing. **Reem Abumallouh**, **Natalia Marin**, **Oihane Alvarez**, and **Carmen Freire:** data curation; funding acquisition; writing – review and editing. **Ana Molina‐Barceló** and **Mercedes Vanaclocha‐Espí:** data curation; writing – review and editing. **Ferran Ballester:** supervision; methodology; writing – review and editing. **Ana Esplugues:** conceptualization; methodology; writing – review and editing. **Maria‐Jose Lopez‐Espinosa:** conceptualization; data curation; formal analysis; funding acquisition; investigation; methodology; supervision; writing – original draft; writing – review and editing.

## Funding

This work was primarily supported by the Ministry of Science, Innovation and Universities (Grant CNS2023‐145286, funded by MICIU/AEI/10.13039/501100011033 and by the European Union NextGenerationEU/PRTR). Additional European funding was provided through the ATHLETE project (Grant Agreement No. 874583) under the Horizon 2020 programme and the JAPreventNCD project (Grant Agreement No. 101128023), co‐funded by the EU4Health Programme 2021–2027. Further support was provided by Spanish funding bodies, including the Fundación Científica Asociación Española Contra el Cáncer (MICROVAGIPAP: IDEAS19098LOPE); the CIBER of Epidemiology and Public Health (PAPILONGO: ESP21PI03); the General Council of Official Nursing Associations of Spain (PAPISEX: inv_cge_2022_04); the Foundation for the Promotion of Health and Biomedical Research of the Valencian Region (PAPILONG: UGP‐20‐242); the Regional Ministry of Innovation, Universities, Science and Digital Society of the Generalitat Valenciana (MENTABIOTA: AICO/2021/182 and CIAICO/2023/184); and Fundació La Marató (202414). In addition, this article was supported by funding for research positions from the Carlos III Health Institute (Miguel Servet‐FEDER: CP11/00178, MSII16/00051, CP20/0006, and Sara Borrell: CD23/00090, all co‐funded by the European Union), as well as the Regional Ministry of Innovation, Universities, Science and Digital Society (Investigo contracts: INVEST/2022/310, INVEST/2023/219, and CIACIF/2022/268). The latter corresponds to a grant from the Programme for the Promotion of Scientific Research, Technological Development and Innovation in the Valencian Community, supporting the recruitment of predoctoral research staff and eligibility for co‐financing by the European Social Fund.

## Disclosure

Artificial intelligence tools were used solely for minor language support. All content was critically reviewed and approved by the authors, who take full responsibility for the manuscript.

## Ethics Statement

This study was performed in line with the principles of the Declaration of Helsinki. All study protocols, including two secondary population‐based registries and two datasets collected by the research team, were approved by the Directorate of Public Health and the Higher Centre for Research in Public Health (DGSP‐CSISP) Ethics Committee from Valencia (references numbers: 20180112/03; 20 190 329/3; 20 190 301/09/1; and 20 210 604/10/01). Secondary data were provided anonymized, while investigator collected data were anonymized prior to analysis following written informed consent from participants or legal guardians (for minors).

## Conflicts of Interest

The authors declare no conflicts of interest.

## Supporting information


**Table S1:** Number of participants in the initial study population (*N* = 417 260) by year of birth.
**Table S2:** Reported country of birth per continent.
**Table S3:** Available determinant data for the Bayesian analysis divided by 5‐year cohort.
**Table S4:** Comparison between included and excluded populations from the Bayesian analyses.
**Table S5:** Descriptive statistics of mean age by 5‐year birth cohort.
**Figure S1:** Flowchart showing the evolution from the initial to the final population sample used in this study.
**Figure S2:** Sensitivity analysis: Comparison of secular trend in age at menarche (years) between the initial population and the population included in the Bayesian analysis.
**Figure S3:** Sensitivity analysis: Comparison of secular trend in age at menarche (years) between population sources.

## Data Availability

Data from population‐based registries (Valencian Breast Cancer Screening Program and the Ambulatory Care Information System of the Valencian Health Agency) are not publicly available due to ethical and legal restrictions related to the use of sensitive health data, and access is restricted. Data from the INMA and PAPILONG cohort studies are available upon request to the corresponding author and in accordance with the applicable data access procedures and ethical approvals. The INMA cohort data are subject to the INMA collaboration policy (https://www.proyectoinma.org/en/inma‐project/inma‐collaborationpolicy/).
