# Supplementary material for: Secular Trend in Age at Menarche and Associated Determinants in the Valencian Population (Spain)
Source: Am J Hum Biol. 2026 Sep 1;38(9):e70322. doi: 10.1002/ajhb.70322 (PMC13535273; doi:10.1002/ajhb.70322)
Supplement: Supplementary file 1 [file AJHB-38-e70322-s001.docx]

**Secular trend in age at menarche and associated determinants in the Valencian population (Spain)**

Andrea Beneito, Blanca Sarzo, Raul Beneyto, Reem Abumallouh, Natalia Marin, Oihane Alvarez, Ana Molina-Barceló, Mercedes Vanaclocha-Espí, Carmen Freire, Ferran Ballester, Ana Esplugues, Maria-Jose Lopez-Espinosa

**Supporting Information**

**Table S1:** Number of participants in the initial study population (N = 417,260**)** by year of birth

**Table S2:** Reported country of birth per continent

**Table S3:** Available determinant data for the Bayesian analysis divided by 5-year cohort

**Table S4:** Comparison between included and excluded populations from the Bayesian analyses

**Table S5:** Descriptive statistics of mean age by 5-year birth cohort

**Figure S1:** Flowchart showing the evolution from the initial to the final population sample used in this study

**Figure S2:** Sensitivity analysis: Comparison of secular trend in age at menarche (years) between the initial population and the population included in the Bayesian analysis

**Figure S3:** Sensitivity analysis: Comparison of secular trend in age at menarche (years) between population sources

**Table S1.** Number of participants in the initial study population (N = 417,260**)** by year of birth

| **Year** | **1931** | **1932** | **1933** | **1934** | **1935** | **1936** | **1937** | **1938** | **1939** | **1940** |
| --- | --- | --- | --- | --- | --- | --- | --- | --- | --- | --- |
| **N** | 92 | 413 | 1,251 | 2,358 | 3,712 | 3,404 | 1,518 | 1,401 | 1,283 | 2,253 |
| **Year** | **1941** | **1942** | **1943** | **1944** | **1945** | **1946** | **1947** | **1948** | **1949** | **1950** |
| **N** | 1,855 | 2,174 | 2,579 | 2,742 | 2,973 | 3,061 | 3,438 | 3,900 | 4,020 | 4,394 |
| **Year** | **1951** | **1952** | **1953** | **1954** | **1955** | **1956** | **1957** | **1958** | **1959** | **1960** |
| **N** | 4,852 | 8,118 | 14,716 | 18,637 | 19,486 | 19,425 | 20,044 | 21,473 | 21,414 | 22,277 |
| **Year** | **1961** | **1962** | **1963** | **1964** | **1965** | **1966** | **1967** | **1968** | **1969** | **1970** |
| **N** | 21,733 | 18,672 | 16,164 | 17,185 | 14,921 | 21,926 | 12,659 | 11,783 | 9,811 | 10,382 |
| **Year** | **1971** | **1972** | **1973** | **1974** | **1975** | **1976** | **1977** | **1978** | **1979** | **1980** |
| **N** | 7,820 | 8,537 | 5,667 | 109 | 30 | 22 | 22 | 15 | 4 | 10 |
| **Year** | **1981** | **1982** | **1983** | **1984** | **1985** | **1986** | **1987** | **1988** | **1989** | **1990** |
| **N** | 3 | 7 | 1 | 0 | 1 | 0 | 0 | 0 | 0 | 51 |
| **Year** | **1991** | **1992** | **1993** | **1994** | **1995** | **1996** | **1997** | **1998** | **1999** | **2000** |
| **N** | 229 | 454 | 758 | 1,129 | 1,196 | 1,386 | 1,403 | 1,421 | 1,595 | 1,589 |
| **Year** | **2001** | **2002** | **2003** | **2004** | **2005** | **2006** | **2007** | **2008** |  |  |
| **N** | 1,539 | 1,531 | 1,553 | 1,637 | 1,589 | 1,036 | 405 | 12 |  |  |

Note: N: sample size

**Table S2.** Reported country of birth per continent

| **Reported country** | **Continent** | **Participants born 1931–1985 (N)^b^** | **Participants born 1990–2008^a^ (N)^b^** |
| --- | --- | --- | --- |
|  | **Europe** | |  |
| Albania |  | 3 | 0 |
| Andorra |  | 8 | 0 |
| Austria |  | 34 | 1 |
| Belarus |  | 21 | 0 |
| Belgium |  | 490 | 6 |
| Bosnia-Herzegovina |  | 17 | 0 |
| Bulgaria |  | 1,178 | 46 |
| Czechoslovakia |  | 13 | 0 |
| Czech Republic |  | 27 | 1 |
| Croatia |  | 11 | 0 |
| Denmark |  | 31 | 0 |
| Estonia |  | 11 | 1 |
| Finland |  | 39 | 0 |
| France |  | 4,475 | 8 |
| Germany |  | 1,311 | 9 |
| Greece |  | 4 | 1 |
| Hungary |  | 40 | 0 |
| Iceland |  | 5 | 0 |
| Ireland |  | 88 | 1 |
| Italy |  | 286 | 20 |
| Latvia |  | 35 | 2 |
| Lithuania |  | 235 | 7 |
| Luxembourg |  | 12 | 0 |
| Malta |  | 4 | 0 |
| Moldova |  | 167 | 2 |
| Monaco |  | 7 | 0 |
| Montenegro |  | 1 | 0 |
| The Netherlands |  | 427 | 5 |
| North Macedonia |  | 3 | 0 |
| Norway |  | 106 | 4 |
| Poland |  | 244 | 11 |
| Portugal |  | 243 | 3 |
| Romania |  | 4,176 | 133 |
| Russia |  | 706 | 12 |
| Russian Federation |  | 5 | 0 |
| Serbia |  | 2 | 0 |
| Slovakia |  | 25 | 0 |
| Slovenia |  | 11 | 0 |
| Spain |  | 163,711 | 11,704 |
| Sweden |  | 101 | 1 |
| Switzerland |  | 469 | 4 |
| United Kingdom |  | 3,053 | 60 |
| Ukraine |  | 1,029 | 28 |
| Vatican City |  | 4 | 0 |
| Yugoslavia |  | 16 | 5 |
| Total (91.0%) |  | 182,884 (90.9%) | 12,075 (93.4%) |
|  | **South/Central America** | |  |
| Argentina |  | 1,820 | 68 |
| Aruba |  | 1 | 0 |
| Bahamas |  | 2 | 0 |
| Bolivia |  | 1,035 | 140 |
| Brazil |  | 632 | 16 |
| Chile |  | 333 | 14 |
| Colombia |  | 3,684 | 153 |
| Costa Rica |  | 15 | 0 |
| Cuba |  | 661 | 12 |
| Dominica |  | 5 | 0 |
| Dominican Republic |  | 341 | 9 |
| Ecuador |  | 2,654 | 131 |
| El Salvador |  | 27 | 2 |
| Grenada |  | 1 | 0 |
| Guatemala |  | 17 | 0 |
| Honduras |  | 158 | 18 |
| Jamaica |  | 3 | 0 |
| Mexico |  | 160 | 3 |
| Netherlands Antilles |  | 3 | 0 |
| Nicaragua |  | 37 | 3 |
| Panama |  | 14 | 2 |
| Paraguay |  | 272 | 13 |
| Peru |  | 524 | 9 |
| Puerto Rico |  | 4 | 0 |
| South America (unspecified) |  | 1 | 0 |
| Uruguay |  | 740 | 30 |
| Venezuela |  | 762 | 49 |
| Total (6.89%) |  | 14,070 (6.99%) | 685 (5.30%) |
|  | **Africa** | |  |
| Africa (unspecified) |  | 13 | 0 |
| Algeria |  | 408 | 5 |
| Angola |  | 9 | 0 |
| Benin |  | 1 | 0 |
| Burkina Faso |  | 1 | 1 |
| Cameroon |  | 11 | 3 |
| Central African Republic |  | 1 | 0 |
| Chad |  | 1 | 0 |
| Congo |  | 12 | 0 |
| Cape Verde |  | 18 | 0 |
| Egypt |  | 5 | 1 |
| Ethiopia |  | 5 | 2 |
| Equatorial Guinea |  | 121 | 15 |
| Gambia |  | 1 | 0 |
| Ghana |  | 2 | 2 |
| Guinea |  | 35 | 1 |
| Guinea-Bissau |  | 1 | 0 |
| Ivory Coast |  | 5 | 0 |
| Kenya |  | 2 | 0 |
| Lesotho |  | 1 | 0 |
| Liberia |  | 2 | 0 |
| Libya |  | 2 | 0 |
| Mali |  | 3 | 0 |
| Mauritius |  | 2 | 0 |
| Mauritania |  | 6 | 1 |
| Morocco |  | 2,227 | 51 |
| Mozambique |  | 4 | 0 |
| Nigeria |  | 24 | 6 |
| Rhodesia |  | 1 | 0 |
| São Tomé and Príncipe |  | 1 | 0 |
| Senegal |  | 50 | 2 |
| Sierra Leone |  | 1 | 0 |
| South Africa |  | 15 | 0 |
| Swaziland |  | 1 | 0 |
| Tanzania |  | 2 | 0 |
| Tunisia |  | 13 | 1 |
| Zaire |  | 9 | 0 |
| Zimbabwe |  | 2 | 0 |
| Total (1.45%) |  | 3,009 (1.50%) | 92 (0.71%) |
|  | **Asia** |  |  |
| Afghanistan |  | 3 | 0 |
| Armenia |  | 195 | 14 |
| Azerbaijan |  | 7 | 0 |
| Bahrain |  | 1 | 0 |
| Bangladesh |  | 1 | 0 |
| Myanmar |  | 1 | 0 |
| Brunei |  | 2 | 0 |
| Cambodia |  | 1 | 0 |
| China |  | 448 | 33 |
| Georgia |  | 71 | 0 |
| India |  | 35 | 3 |
| Indonesia |  | 7 | 0 |
| Iraq |  | 5 | 0 |
| Iran |  | 24 | 1 |
| Japan |  | 24 | 0 |
| Jordan |  | 3 | 0 |
| Kazakhstan |  | 10 | 1 |
| Kuwait |  | 1 | 0 |
| Kyrgyzstan |  | 3 | 0 |
| Laos |  | 4 | 0 |
| Lebanon |  | 9 | 0 |
| Malaysia |  | 7 | 0 |
| Mongolia |  | 1 | 0 |
| Nepal |  | 4 | 0 |
| North Korea |  | 4 | 0 |
| Pakistan |  | 65 | 12 |
| Palestine |  | 1 | 0 |
| Philippines |  | 78 | 0 |
| Saudi Arabia |  | 1 | 1 |
| Singapore |  | 3 | 0 |
| South Korea |  | 4 | 0 |
| South Vietnam |  | 1 | 0 |
| Sri Lanka |  | 1 | 0 |
| Syria |  | 28 | 5 |
| Taiwan |  | 5 | 0 |
| Thailand |  | 15 | 0 |
| Turkey |  | 4 | 0 |
| United Arab Emirates |  | 0 | 1 |
| Uzbekistan |  | 5 | 0 |
| Vietnam |  | 8 | 0 |
| Total (0.54%) |  | 1,094 (0.54%) | 71 (0.55%) |
|  | **North America & Oceania** | | |
| Australia |  | 26 | 0 |
| Canada |  | 30 | 1 |
| North America (unspecified) |  | 3 | 0 |
| United States of America |  | 152 | 4 |
| Total (0.10%) |  | 211 (0.10%) | 5 (0.04%) |
| Total |  | 201,268 | 12,928 |

Note: ^a^For the 1986–1990 birth cohort, all participants were born in 1990. ^b^Sample size (N) is presented for individual countries; totals are presented as N (%).

Participants who reported African, North American or South American origin without specifying country of origin were grouped in the ‘Africa (unspecified)’ (N = 13), ‘North America (unspecified)’ (N = 3) or ‘South America (unspecified)’ (N = 1) category. North America and Oceania were combined due to low numbers.

| **5-year birth cohort** | Initial sample size^a^ | Continent of birth^b^ | Educational level^b^ | BMI (z-score)^b^ | Final sample size^c^ |
| --- | --- | --- | --- | --- | --- |
|  | **Participants born 1931–1985** | | | | |
| **1931–1935** | 7,826 | **–** | 7,202 (92.0) | **–** | **–** |
| **1936–1940** | 9,859 | 6 (0.06) | 7,697 (78.1) | **–** | **–** |
| **1941–1945** | 12,323 | 515 (4.18) | 9,099 (73.8) | **–** | 472 (3.83) |
| **1946–1950** | 18,813 | 2,409 (12.8) | 14,983 (79.6) | **–** | 2,099 (11.2) |
| **1951–1955** | 65,809 | 14,467 (22.0) | 55,382 (84.2) | **–** | 13,037 (19.8) |
| **1956–1960** | 104,633 | 25,334 (24.2) | 86,789 (82.9) | **–** | 22,759 (21.8) |
| **1961–1965** | 88,675 | 70,904 (80.0) | 81,232 (91.6) | **–** | 66,014 (74.4) |
| **1966–1970** | 66,561 | 65,564 (98.5) | 57,852 (86.9) | **–** | 56,677 (85.2) |
| **1971–1975** | 22,163 | 21,858 (98.6) | 16,294 (73.5) | **–** | 15,967 (72.0) |
| **1976–1980** | 73 | 73 (100) | 73 (100) | **–** | **–** |
| **1981–1985** | 12 | 12 (100) | 12 (100) | **–** | **–** |
|  | **Participants born 1990–2008^d,e^** | | | | |
| **1986–1990^d^** | 51 | 30 (58.8) | **–** | 48 (94.1) | **–** |
| **1991–1995** | 3,766 | 2919 (77.5) | **–** | 3,533 (93.8) | 2,710 (72.0) |
| **1996–2000** | 7,394 | 5710 (77.2) | **–** | 7,144 (96.6) | 5,463 (73.9) |
| **2001–2005** | 7,849 | 3887 (49.5) | **–** | 7,685 (97.9) | 3,694 (47.1) |
| **2006–2010^e^** | 1,453 | 370 (25.5) | **–** | 1,433 (98.6) | **–** |

**Table S3**. Available determinant data for the Bayesian analysis divided by 5-year cohort

Note: ^a^Initial sample size of the population included in each birth cohort. ^b^Number (%) of participants with available information on the variable of interest, with percentages calculated relative to the initial sample size. For the analytical sample of continent of birth, participants born in North America, Oceania, and Asia (both analyses), and Africa (participants born 1990**–**2008 only), were excluded because of small sample size. ^c^Number (%) of participants with complete information on year of birth, continent of birth and educational level (participants born 1931**–**1985), or year of birth, continent of birth and body mass index (participants born 1990**–**2008), included in the Bayesian analyses of determinants, with percentages calculated relative to the initial sample size. ^d^For the 1986–1990 birth cohort, all participants were born in 1990. ^e^For the most recent 5-year birth cohort (2006–2010), data were available only for births up to 2008. BMI: body mass index, based on WHO age- and sex-specific z-scores (De Onis et al., 2007) and classified as low (≤–2 standard deviations [SD]), normal (>–2 to ≤1 SD), or high (>1 SD). Sample size is presented as N and categorical variables as N (%).

**Table S4.** Comparison between included and excluded populations from the Bayesian analyses

| **Variable** | **Included**  **(N = 188,892)** | **Excluded**  **(N = 228,368)** | *p-value^a^* |
| --- | --- | --- | --- |
| Age at menarche (years) | 12.6 ± 1.57 | 12.6 ± 1.57 | 0.111 |
| Continent of birth^b,c^ |  |  |  |
| Europe | 174,622 (92.4) | 20,337 (80.8) | < 0.001 |
| South/Central America | 11,945 (6.32) | 2,669 (10.6) |  |
| Africa | 2,325 (1.23) | 784 (3.12) |  |
| Asia | 0 (0) | 1,160 (4.61) |  |
| North America/Oceania | 0 (0) | 216 (0.96) |  |
| Educational level^b^ |  |  |  |
| Primary education | 92,825 (52.4) | 116,911 (73.3) | < 0.001 |
| Secondary education | 51,090 (28.9) | 23,828 (14.9) |  |
| University education | 33,110 (18.7) | 18,851 (11.8) |  |
| BMI (z-score)^c^ |  |  |  |
| Low BMI | 110 (0.93) | 82 (1.03) | 0.028 |
| Normal BMI | 6,683 (56.3) | 4,345 (54.5) |  |
| High BMI | 5,074 (42.8) | 3,549 (44.5) |  |

Note: ^a^*p*-*values* obtained by comparing both groups using logistic regression models and the likelihood ratio test (LRT); ^b^Variables included in the model for participants born between 1941 and 1975; ^c^Variables included in the model for participants born between 1991 and 2005. BMI: body mass index, based on WHO age- and sex-specific z-scores (De Onis et al., 2007) and classified as low (≤–2 standard deviations [SD]), normal (>–2 to ≤1 SD), or high (>1 SD).

Categorical variables are presented as N (%) and continuous variables as mean ± SD. The included population was derived from the total study population described in Table 1 and restricted to participants with complete data on all the determinants studied included in the Bayesian analyses.

**Table S5.** Descriptive statistics of mean age by 5-year birth cohort

|  | **Population included in the temporal series** | | |  | **Population included in the Bayesian analyses^a^** | | |
| --- | --- | --- | --- | --- | --- | --- | --- |
| **5-year birth cohort** | **Sample size** | **Age at menarche (years)** | |  | **Sample size** | **Age at menarche (years)** | |
|  | **N** | **Mean ± SD** | **Median (P25, P75)** |  | **N** | **Mean ± SD** | **Median (P25, P75)** |
|  | **Participants born 1931–1985 (N = 396,747)** | | |  | **Participants born 1941–1975: earlier-born group (N = 177,025)** | | |
| **1931–1935** | 7,826 | 13.1 ± 1.65 | 13 (12, 14) |  | 0 | **–** | **–** |
| **1936–1940** | 9,859 | 12.9 ± 1.67 | 13 (12, 14) |  | 0 | **–** | **–** |
| **1941–1945** | 12,323 | 12.7 ± 1.64 | 13 (12, 14) |  | 472 | 12.8 ± 1.57 | 13 (12, 14) |
| **1946–1950** | 18,813 | 12.6 ± 1.59 | 13 (12, 14) |  | 2,099 | 12.8 ± 1.61 | 13 (12, 14) |
| **1951–1955** | 65,809 | 12.6 ± 1.55 | 13 (11, 14) |  | 13,037 | 12.6 ± 1.58 | 13 (12, 14) |
| **1956–1960** | 104,633 | 12.5 ± 1.56 | 13 (11, 14) |  | 22,759 | 12.6 ± 1.58 | 13 (11, 14) |
| **1961–1965** | 88,675 | 12.7 ± 1.56 | 13 (12, 14) |  | 66,014 | 12.6 ± 1.56 | 13 (12, 14) |
| **1966–1970** | 66,561 | 12.7 ± 1.58 | 13 (12, 14) |  | 56,677 | 12.7 ± 1.57 | 13 (12, 14) |
| **1971–1975** | 22,163 | 12.7 ± 1.59 | 13 (12, 14) |  | 15,967 | 12.7 ± 1.59 | 13 (12, 14) |
| **1976–1980** | 73 | 12.3 ± 1.70 | 13 (11, 13) |  | 0 | **–** | **–** |
| **1981–1985** | 12 | 12.3 ± 1.60 | 12 (11, 13) |  | 0 | **–** | **–** |
|  | **Participants born 1990–2008^b,c^ (N = 20,513)** | | |  | **Participants born 1991–2005: later-born group (N = 11,867)** | | |
| **1986–1990^b^** | 51 | 12.2 ± 1.57 | 12 (11, 13) |  | 0 | **–** | **–** |
| **1991–1995** | 3,766 | 12.0 ± 1.35 | 12 (11, 13) |  | 2,710 | 12.0 ± 1.34 | 12 (11, 13) |
| **1996–2000** | 7,394 | 11.7 ± 1.18 | 12 (11, 13) |  | 5,463 | 11.7 ± 1.18 | 12 (11, 12) |
| **2001–2005** | 7,849 | 11.6 ± 1.10 | 12 (11, 12) |  | 3,694 | 11.6 ± 1.10 | 12 (11, 12) |
| **2006–2010^c^** | 1,453 | 11.1 ± 0.84 | 11 (11, 12) |  | 0 | **–** | **–** |

Note: ^a^Bayesian analyses of the determinants associated with the secular trend of menarche were conducted in a subsample, including only participants with complete data on all the studied determinants and excluding: 1) participants with incomplete data on any of the determinants under study; 2) birth cohorts 1931–1940, 1976–1990, and 2006–2010 (the last truncated at 2008 due to data being unavailable) because of small sample sizes with determinant information; and 3) participants born in North America, Oceania, Asia (analyses for both groups) and Africa (analyses for the later-born group) due to small sample sizes. **^b^**For the 1986–1990 birth cohort, all participants were born in 1990. ^c^For the most recent 5-year birth cohort (2006–2010), data availability was limited to births up to 2008. Age at menarche is presented as mean ± standard deviation (SD) and median (25th–75th percentiles, P25–P75).

**Figure S1.** Flowchart showing the evolution from the initial to the final population sample used in this study

Note: Two populations were used in the present study: one for the temporal series and another for the Bayesian analyses. Bayesian analyses of the determinants associated with the secular trend of menarche were conducted in a subsample, including only participants with complete data on all the studied determinants and excluding birth cohorts 1931–1940, 1976–1990, and 2006–2008 (the last truncated at 2008 due to data being unavailable) because of small sample sizes with determinant information, and excluding participants born in North America, Oceania, Asia (analyses of both birth groups), and Africa (analyses of the later-born group) owing to small sample sizes. Separate Bayesian linear regression models were fitted for participants born 1941–1975 (earlier-born group) and those born 1991–2005 (later-born group). Models were adjusted for birth cohort and continent of birth, with additional adjustment for educational level in the earlier-born group and body mass index (BMI) in the later-born group. Although birth cohorts were defined as 5-year intervals in this study, only the birth years for which data were available (1990–2008) are represented in this figure. Consequently, the 1986–1990 birth cohort included only births from 1990, whereas the 2006–2010 birth cohort included only births up to 2008.

**Figure S2.** Sensitivity analysis: Comparison of secular trend in age at menarche (years) between the initial population and the population included in the Bayesian analysis

Note: N: sample size. Red line: participants included in the temporal series analysis, born between 1931 and 2008. For the most recent 5-year birth cohort (2006–2010), data were available only for births up to 2008. Blue line: subset of the study population included in the Bayesian analysis, including participants born between 1941 and 1975 and those born between 1991 and 2005.

**Figure S3.** Sensitivity analysis: Comparison of secular trend in age at menarche (years) between population sources

Note: Red line: participants from the Valencian Breast Cancer Screening Program (BCSP). Yellow line: participants from the PAPILONG cohort. Green line: participants from the INMA cohort (mothers). Blue line: participants from the INMA cohort (girls). Purple line: participants from the Ambulatory Care Information System of the Valencian Health Agency (SIA-GAIA). For the most recent 5-year birth cohort (2006–2010), data availability was limited to births up to 2008.

**References**

De Onis, M., Onyango, A. W., Borghi, E., Siyam, A., Nishida, C., & Siekmann, J. (2007). Development of a WHO growth reference for school-aged children and adolescents. *Bulletin of the World Health Organization*, *85*(9), 660–667. https://doi.org/10.2471/BLT.07.043497
